# Introduction to serial reviews: Current progress in covalent modification of biomolecules by compounds in food or oxidatively generated compounds - its relevance to biological functions

**DOI:** 10.3164/jcbn.17-200

**Published:** 2017-12-12

**Authors:** Yoji Kato

**Affiliations:** 1 Laboratory of Free Radical and Food Function, School of Human Science and Environment, University of Hyogo, 1-1-12 Shinzaike-honcho, Himeji, Hyogo 670-0092, Japan; 2 Research Institute for Food and Nutrition, University of Hyogo, 1-1-12 Shinzaike-honcho, Himeji, Hyogo 670-0092, Japan

As a consequence of oxidative stress, lipids, proteins, and DNA are endogenously oxidized, and this leads to the formation of modified molecules in tissue and blood.^(1–3)^ Some of these products are excreted in urine. Among these oxidized molecules, lipid peroxidation-derived products (aldehydes) or quinones are highly reactive and form adducts with amine moieties in DNA and amine/thiol moieties in proteins.^(3–9)^ Oxidative stress is considered to be caused by and be a result of disease. Therefore, oxidized molecules could be factors in the initiation, promotion, and development of pathologies such as cardiovascular^(10)^ and neurodegenerative diseases.^(11)^ Therefore, the detection of these molecules could serve as a “torch” for researchers to reveal the picture of such diseases. These modified molecules are a kind of “rust” in living systems and could be indicators of a balance between oxidation and reduction.

We have food every day. Food contains nutrients (proteins, sugars and lipids). Plant-derived food, in particular, is also rich in non-nutrients such as phytochemicals (e.g., polyphenols). This indicates that “xenobiotics”, which are not true nutrients, are incorporated into our daily diets whether we like it or not. Nutritionally, we do not “digest” these xenobiotics (e.g., phytochemicals), but some are circulated through the body either intact or in conjugated forms. These ingested phytochemicals could elicit biological effects. Indeed, epidemiological studies have shown the beneficial effect of polyphenols in, for example, the prevention of cardiovascular diseases.^(12)^ These effects may be triggered by specific or non-specific reactions between ingested xenobiotics and various biological molecules within cells.^(13)^ Plant-derived isothocyanates can form adducts with proteins and thereby induce cellular responses,^(14)^ while some polyphenols can conjugate with proteins via their quinone moieties.^(15)^ These xenobiotics may act as “toxic chemicals” that induce self-defense mechanisms in the body.^(16–17)^ Alternatively, some biological effects could be caused by “indirect” reactions between a phytochemical and a cell via a receptor.^(18)^ This means that the “brain-gut interaction” is a pathway to instantiate functions triggered by the ingestion of fruits and vegetables.

In these serial reviews, current progress in covalent modification of biomolecules by food or oxidatively generated compounds will be reviewed in six separate papers from different points of view. In particular, these reviews will focus on the biological relevance of the interaction of oxidized or ingested molecules with biomolecules and their role in triggering biological functions via shared pathways (Fig. 1).

## Figures and Tables

**Fig. 1:**
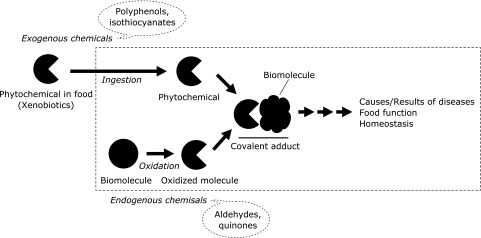
Schematic illustration of two-into-one pathway for covalent adduction that could contribute biological function. Food-derived ingested chemicals and endogenously generated oxidized products may share the pathway for the expression of biological function.
